# A framework of biomarkers for skeletal aging: a consensus statement by the Aging Biomarker Consortium

**DOI:** 10.1093/lifemedi/lnad045

**Published:** 2023-11-22

**Authors:** Aging Biomarker Consortium, Jinlong Suo, Yibo Gan, Yangli Xie, Shuqin Xu, Jianfang Wang, Di Chen, Lin Chen, Lianfu Deng, Shiqing Feng, Jingdong Jackie Han, Qing Jiang, Guanghua Lei, Peng Liu, Xianghang Luo, Xin Ma, Jing Qu, Chunli Song, Peifu Tang, Tingting Tang, Sijia Wang, Xiaochun Wei, Chengtie Wu, Guozhi Xiao, Liu Yang, Licheng Zhang, Weiqi Zhang, Zhenlin Zhang, Guang-Hui Liu, Changqing Zhang, Gang Pei, Jian Luo, Rui Yue, Weiguo Zou

**Affiliations:** Department of Orthopedic Surgery, Institute of Microsurgery on Extremities, Shanghai Sixth People’s Hospital Affiliated to Shanghai Jiao Tong University School of Medicine, Shanghai, China; Department of Orthopedic Surgery, Institute of Microsurgery on Extremities, Shanghai Sixth People’s Hospital Affiliated to Shanghai Jiao Tong University School of Medicine, Shanghai, China; State Key Laboratory of Trauma and Chemical Poisoning, Department of Spine Surgery, Center of Orthopedics, Daping Hospital, Army Medical University (Third Military Medical University), Chongqing, China; State Key Laboratory of Trauma and Chemical Poisoning, Department of Wound Repair and Rehabilitation Medicine, Center of Bone Metabolism and Repair (CBMR), Daping Hospital, Army Medical University (Third Military Medical University), Chongqing, China; Department of Osteoporosis and Bone Diseases, Shanghai Clinical Research Center of Bone Disease, Shanghai Sixth People’s Hospital Affiliated to Shanghai Jiao Tong University School of Medicine, Shanghai, China; Shanghai Key Laboratory of Signaling and Disease Research, Frontier Science Center for Stem Cell Research, School of Life Sciences and Technology, Institute for Regenerative Medicine, Shanghai East Hospital, Tongji University, Shanghai, China; Research Center for Computer-Aided Drug Discovery, Faculty of Pharmaceutical Sciences, Shenzhen Institute of Advanced Technology, Chinese Academy of Sciences, Shenzhen, China; State Key Laboratory of Trauma and Chemical Poisoning, Department of Wound Repair and Rehabilitation Medicine, Center of Bone Metabolism and Repair (CBMR), Daping Hospital, Army Medical University (Third Military Medical University), Chongqing, China; Shanghai Key Laboratory for Prevention and Treatment of Bone and Joint Diseases, Department of Orthopaedics, Shanghai Institute of Traumatology and Orthopaedics, Ruijin Hospital, Shanghai Jiao Tong University School of Medicine, Shanghai, China; Department of Orthopaedics, Qilu Hospital of Shandong University, Shandong University Centre for Orthopaedics, Advanced Medical Research Institute, Cheeloo College of Medicine, Shandong University, Jinan, China; Peking-Tsinghua Center for Life Sciences, Academy for Advanced Interdisciplinary Studies, Center for Quantitative Biology (CQB), Peking University, Beijing, China; State Key Laboratory of Pharmaceutical Biotechnology, Division of Sports Medicine and Adult Reconstructive Surgery, Department of Orthopedic Surgery, Drum Tower Hospital affiliated to Medical School of Nanjing University, Nanjing, China; Key Laboratory of Aging-related Bone and Joint Diseases Prevention and Treatment, Ministry of Education, Hunan Key Laboratory of Joint Degeneration and Injury, Department of Orthopaedics, National Clinical Research Center for Geriatric Disorders, Xiangya Hospital, Central South University, Changsha, China; State Key Laboratory of Trauma and Chemical Poisoning, Department of Spine Surgery, Center of Orthopedics, Daping Hospital, Army Medical University (Third Military Medical University), Chongqing, China; Key Laboratory of Aging-related Bone and Joint Diseases Prevention and Treatment, Ministry of Education, Key Laboratory of Organ Injury, Aging and Regenerative Medicine of Hunan Province, Department of Endocrinology, Endocrinology Research Center, National Clinical Research Center for Geriatric Disorders, Xiangya Hospital, Central South University, Changsha, China; Department of Orthopaedic Surgery, Shanghai Sixth People’s Hospital Affiliated to Shanghai Jiao Tong University School of Medicine, Shanghai, China; State Key Laboratory of Stem Cell and Reproductive Biology, Institute of Zoology, Institute for Stem Cell and Regeneration, Institute for Stem Cell and Regenerative Medicine, University of Chinese Academy of Sciences, Beijing, China; Beijing Key Laboratory of Spinal Disease, Department of Orthopedics, Engineering Research Center of Bone and Joint Precision Medicine, Peking University Third Hospital, Beijing, China; Department of Orthopaedic Trauma, the Fourth Medical Center, National Clinical Research Center for Orthopaedics & Sports Rehabilitation in China, Chinese PLA General Hospital, Beijing, China; Shanghai Key Laboratory of Orthopaedic Implants, Department of Orthopaedic Surgery, Shanghai Ninth People’s Hospital, Shanghai Jiao Tong University School of Medicine, Shanghai, China; CAS Key Laboratory of Computational Biology, Shanghai Institute of Nutrition and Health, University of Chinese Academy of Sciences, Chinese Academy of Sciences, Shanghai, China; Shanxi Key Laboratory of Bone and Soft Tissue Injury Repair, Department of Orthopedics, the Second Hospital of Shanxi Medical University, Taiyuan, China; State Key Laboratory of High Performance Ceramics and Superfine Microstructure, Shanghai Institute of Ceramics, Chinese Academy of Sciences, Shanghai, China; Guangdong Provincial Key Laboratory of Cell Microenvironment and Disease Research, Shenzhen Key Laboratory of Cell Microenvironment, Department of Biochemistry, School of Medicine, Southern University of Science and Technology, Shenzhen, China; Institute of Orthopedic Surgery, Xijing Hospital, The Fourth Military Medical University, Xi’an, Shaanxi, China; Medical Research Institute, Northwestern Polytechnical University, Xi’an, China; Department of Orthopaedic Trauma, the Fourth Medical Center, National Clinical Research Center for Orthopaedics & Sports Rehabilitation in China, Chinese PLA General Hospital, Beijing, China; CAS Key Laboratory of Genomic and Precision Medicine, Beijing Institute of Genomics, Chinese Academy of Sciences and China National Center for Bioinformation, Beijing, China; Department of Osteoporosis and Bone Diseases, Shanghai Clinical Research Center of Bone Disease, Shanghai Sixth People’s Hospital Affiliated to Shanghai Jiao Tong University School of Medicine, Shanghai, China; State Key Laboratory of Membrane Biology, Institute of Zoology, Institute for Stem Cell and Regeneration, University of Chinese Academy of Sciences, Chinese Academy of Sciences, Beijing, China; Department of Orthopedic Surgery, Institute of Microsurgery on Extremities, Shanghai Sixth People’s Hospital Affiliated to Shanghai Jiao Tong University School of Medicine, Shanghai, China; Collaborative Innovation Center for Brain Science, School of Life Science and Technology, Tongji University, Shanghai, China; Yangzhi Rehabilitation Hospital (Shanghai Sunshine Rehabilitation Center), Tongji University School of Medicine, Shanghai, China; Shanghai Key Laboratory of Signaling and Disease Research, Frontier Science Center for Stem Cell Research, School of Life Sciences and Technology, Institute for Regenerative Medicine, Shanghai East Hospital, Tongji University, Shanghai, China; Department of Orthopedic Surgery, Institute of Microsurgery on Extremities, Shanghai Sixth People’s Hospital Affiliated to Shanghai Jiao Tong University School of Medicine, Shanghai, China; State Key Laboratory of Cell Biology, CAS Center for Excellence in Molecular Cell Sciences, Shanghai Institute of Biochemistry and Cell Biology, Chinese Academy of Sciences, University of Chinese Academy of Sciences, Shanghai, 200031, China

## Abstract

The skeleton is an important structural and metabolic organ in human body, while aging is the physiological basis for degenerative skeletal diseases. China has the largest aging population in the world and faces great challenges in preventing and managing diseases related to skeletal aging. To address these challenges, the Aging China Biomarkers Consortium (ABC) has reached an expert consensus on biomarkers of skeletal aging by synthesizing the literature and insights from scientists and clinicians. The consensus provides a comprehensive assessment of biomarkers associated with skeletal aging and proposes a systematic framework that categorizes biomarkers into three dimensions, namely, functional, structural, and humoral dimensions. Within each dimension, the ABC recommended clinical and evidential research-based biomarkers for physiological aging and degenerative pathologies of the skeleton. This expert consensus aims to lay the foundation for future studies to assess the prediction, diagnosis, early warning, and treatment of diseases associated with skeletal aging, with the ultimate goal of improving the skeletal health of elderly populations in China and around the world.

## Introduction

The skeleton, including bones and joints, is an important part of the human locomotor system. It serves important functions such as structural support, postural maintenance, locomotor capacity, protection of internal organs, mineral storage, and metabolic regulation [1]. Bone not only supports movement but also functions as an endocrine organ. In addition, bone and bone marrow are inextricably linked, and are even considered to be two parts of a single organ. The skeletal system also plays important functions in hematopoiesis and metabolism [2]. Skeletal aging refers to the gradual structural and functional degeneration of bone and joint tissues with age, which is mainly manifested by the depletion of skeletal system-related stem cells, loss of bone mass, alteration of bone microarchitecture, reduction of bone strength, degeneration of cartilage, and sclerosis of subchondral bone [3–5].

Skeletal aging in a broad sense includes not only bone and joint aging, but also disc degeneration, ligament calcification, synovial tissue hypertrophy, and vascular anomalies [6–8]. Skeletal aging is primarily manifested clinically by a high prevalence of multiple degenerative motor system disorders, including osteoporosis, fragility fractures, osteoarthritis (OA), lumbar disc herniation, cervical spondylosis, and scoliosis [9–13]. In addition, skeletal aging may jeopardize the overall health of the organism and is a risk factor for neurological and cardiovascular diseases as well as degenerative diseases such as metabolic syndrome [14–16]. It has even been suggested that bone is a target for anti-aging [17]. Identifying biomarkers of skeletal aging can provide insight into the characteristics of skeletal aging, enable early prediction and diagnosis of skeletal aging, guide personalized therapeutic regimens, and dynamically detect and evaluate the effects of treatments, thereby slowing down the onset and progression of related diseases. Therefore, it is of great clinical value to clarify the biomarkers related to skeletal aging.

On 27 August 2023, the Chinese Aging Biomarker Consortium (ABC) [18, 19] convened a workshop at the Shanghai Sixth People’s Hospital/National Orthopaedic Medical Center to identify the relevant biomarkers for skeletal aging based on the literature and evidence-based research. The expert consensus, based on peer-reviewed studies and evidence-based medicine, recommended biomarkers that can indicate the degree and rate of skeletal aging, predict the risk of skeletal aging-related diseases, and provide a scientific evaluation system for slowing down and intervening in aging and its related diseases. The goal is to slow down aging and offer effective interventions.

## Recommended methodology for biomarkers for skeletal aging

The presentation of the level of recommendation and level of evidence in this consensus follows the internationally accepted approach. At present, the standards for classifying levels of evidence that are widely accepted and used internationally come from the standards for levels of evidence (levels of evidence are classified into three levels, and recommendation suggestions are classified into four levels) formulated by the Oxford Centre for Evidence-based Medicine (OCEM) in 2001 (Table 1).

**Table 1.**
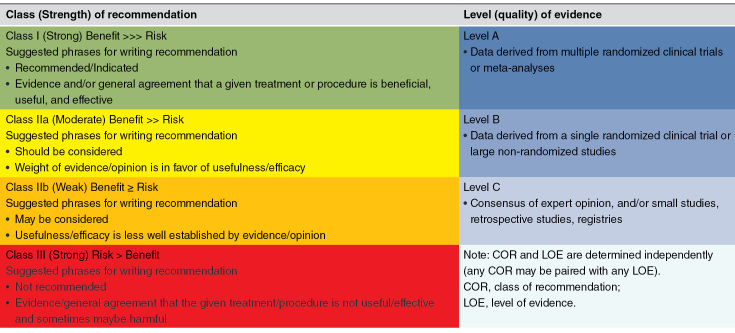
Classification and definition of level of evidence and level of recommendation

**Table 2.**
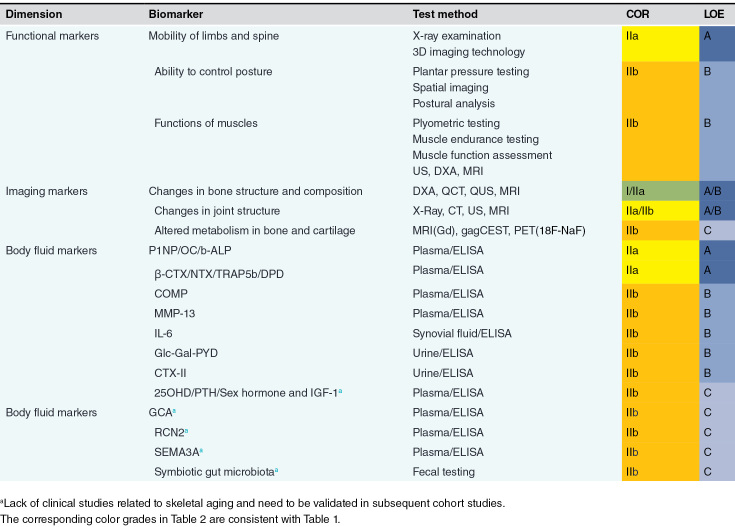
Key recommended biomarkers of skeletal aging

## Classification and clinical application of skeletal aging biomarkers

**Figure 1.**
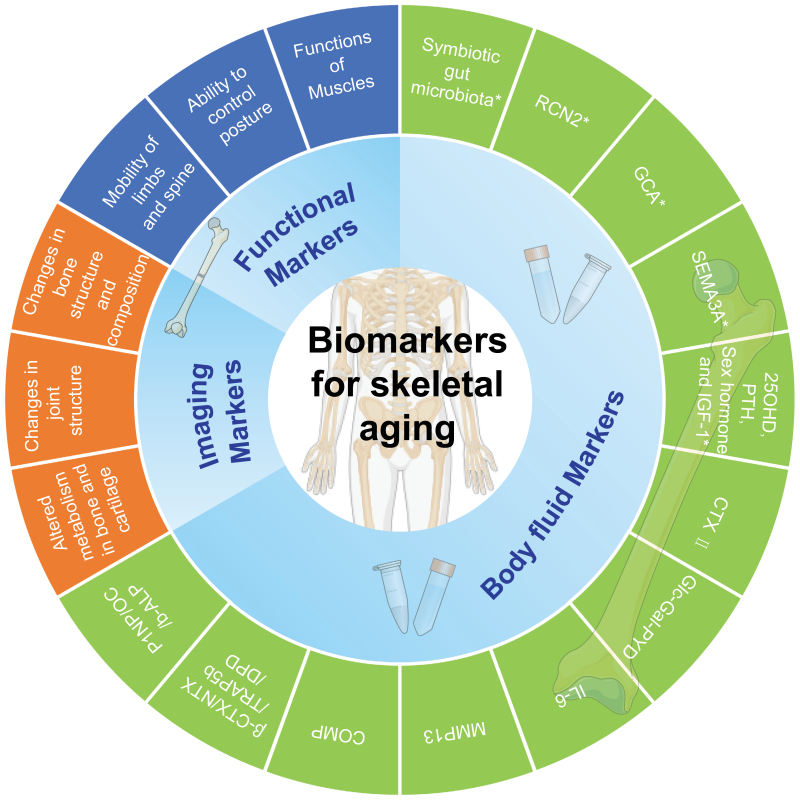
**Summary of proposed recommended markers of skeletal aging.**The proposed framework for the assessment of skeletal aging consists of three dimensions: functional, imaging, and body fluid biomarkers. A wide range of changes occurring at all levels of the skeleton during the aging process are covered. These biomarkers are expected to be widely used in routine clinical practice. However, it is important to emphasize that further validation is needed to assess the validity of these biomarkers in the assessment of skeletal biological aging. Abbreviations involved in the Fig. 1 refer to Table 3. The imaging markers covered in this consensus are relatively specific for skeletal aging, the humoral markers such as P1NP/OC/b-ALP, β-CTX/NTX/TRAP5b/DPD, PTH, are relatively specific for skeletal aging. * Lack of clinical studies related to skeletal aging and need to be validated in subsequent cohort studies.

Skeletal aging involves multidimensional changes in molecules, cells, organs, individuals, and populations [18, 19]. Markers of skeletal aging are indicators that provide an accurate reflection of the actual age, structure, and function of bones and joints. These markers can be used to determine the degree and rate of skeletal aging and assess the risk of disease and the effectiveness of aging interventions. They can be used to determine the degree and speed of bone aging, and to assess the risk of disease and the effectiveness of aging interventions. The present consensus is to screen bone aging markers from three dimensions, namely, bone function, imaging, and body fluids, for reference in clinical work and subsequent studies (Fig. 1).

### Functional markers

#### Limitations of limb and spine movement

The International Classification of Functioning, Disability and Health (ICF) emphasizes that the ability to perform activities of daily living (ADLs) is a key indicator for assessing human health. Common activities include basic movements such as walking, standing, sitting, bending, etc., which depend on good skeletal mobility.

Aging populations generally exhibit reduced mobility [20]. Aging leads to decreased bone density and weakened bone strength, which causes an increased risk of fractures. Fractures in some vital areas such as the hip and spine have a significant negative impact on the body’s mobility functions [21].

Limited joint motion is an important sign of joint aging. Studies have shown that the range of motion (ROM) of most joints in the body decreases with age [22], most commonly in the hip and knee joints. A portion of middle-aged and older adults may develop symptoms of joint stiffness prior to the onset of OA, usually in the morning or after a long period of inactivity. The duration of this symptom is short, usually a few minutes to 10 min, and rarely more than 30 min. With the progression of OA, narrowing of the joint space and the formation of osteophytes can further limit the ROM. In the intermediate stage of the disease, the active and passive ROM of the affected joints can be found through clinical examination or X-ray; in the advanced stage, the restricted joint motion is more severe, and the patient often complains that the restricted joint motion affects the functions of squatting, walking, and other daily life functions. The major cause of joint limitation is that the articular cartilage may gradually wear out and thin with age, resulting in increased friction on the joint surfaces [23]. At the same time, the synovial membrane of the joints may undergo inflammation during the aging process, leading to increased fluid in the joint cavity, joint swelling, and limited joint movement [24]. In addition, decreased function of the muscles surrounding the joints can also lead to limited joint movement.

Limited spinal mobility is also an important feature of skeletal aging. In a healthy state, the spine has better flexibility and stability, enabling it to accomplish multi-directional movements including forward flexion, backward extension, lateral bending, and rotation. Upon aging, the body’s function gradually declines and spinal mobility is limited, which is manifested by decreased spinal ROM, decreased flexibility, decreased rotational ability, and prolonged reaction time [25, 26]. One study found a significant correlation between the inequality and variability of intervertebral angular motion [27], and the ROM of lumbar flexion and extension [28] in patients with age, as evaluated by imaging. A clinical study using a wearable device cervical ROM (CROM) meter to measure cervical spine mobility in 500 healthy people of different ages and genders found that age was negatively correlated to cervical spine mobility, which decreased significantly with age and degeneration of the cervical intervertebral joints [29].

Hence, limb movement limitation can accurately reflect the aging status of the skeleton and is a potential marker of skeletal aging. However, it is important to note that limb mobility also correlates with gender [30], body mass index (BMI) [31], and bone morphology [32]. X-ray examination, combined with a doctor’s diagnosis, is the most common and accurate way to measure bone function in clinical practice. In recent years, new technologies such as 3D imager technology [33], wearable devices [34], and even intelligent cell phones [29] can be used to detect limb mobility more conveniently and visually.

#### Decreased postural control

Postural control is the ability of the human body to maintain or change a particular posture and is accomplished by multiple systems in the body working together. In normal conditions, the body can maintain a variety of movements such as balancing, standing, squatting, and walking through postural control. Postural control is gradually impaired with age, which is an important feature of skeletal aging.

Skeletal aging is closely related to postural control. With skeletal aging, patients with osteoporosis often develop poor balance control and postural deformities [35, 36]. Studies have shown that older patients with osteoporosis have decreased stability in postural control, as evidenced by greater swing speed and center of pressure (COP) displacement, along with postural abnormalities including kyphosis of spine and head fronting [37]. In addition, women with osteoporosis have more pronounced decreases in flexibility and mobility, and are at greater risk for falls than men [38].

Joint degeneration is also strongly associated with reduced postural control in the body [39]. Reduced joint stability, decreased joint ROM, and decreased muscle strength and endurance with age may result in decreased balance, which in turn increases the risk of falls. In addition, decreased joint proprioception may be a risk factor for falls in older adults. A study comparing lower limb joint proprioception and postural balance between young people and older adults found that hip joint proprioception decreased significantly in older adults, as evidenced by a positive correlation between hip joint position sense (JPS) error and the root-mean-square distances of COP in the anterior–posterior direction and medial–lateral direction, which suggests that the decline in hip joint proprioception is closely related to the decline in postural control and balance in older adults. This indicates that the decline in hip joint proprioception is closely related to the decline in postural control and balance ability of the elderly [40].

Postural control is strongly related to spinal aging. On the one hand, the spine gradually loses its stability, elasticity, and load-bearing capacity with age, and suffers from osteoporosis, disc degeneration, vertebral collapse, and other issues, thus affecting the body’s postural control ability. On the other hand, with the decline in postural control of the body, problems such as unstable posture and easy to fall will also exacerbate the degeneration and injury of the spine. It was found that the elderly showed increased center of gravity (COG) swing speed, increased movement time to reach the objective, and increased movement path in standing on one foot with eyes open or closed, which were significantly different from those of middle-aged and young people; this indicates that the elderly have decreased postural control and balance ability [41, 42].

Accordingly, decreased postural control can be a more accurate response to skeletal aging status and is a potential marker of skeletal aging. Postural control can be detected by detecting limb and whole-body COP movement trajectories as well as COG position changes based on plantar pressure testing, spatial imaging, and postural analysis.

#### Muscle function loss

The skeletal and muscular systems both undergo physiological alterations with age, resulting in decreased bone density, strength, joint ROM, and muscle function. Decreased muscle function often couples with bone aging. There is a positive correlation between muscle strength and bone mineral density (BMD) [43]. This is due to the tight interaction between bone and muscle; when a muscle contracts, it exerts a force on the bone, which stimulates growth and remodeling. At the same time, decreased muscle strength can cause a decrease in bone density, leading to an increased risk of fracture [44]. At the same time, bone deterioration affects muscle function [45]. These changes may limit muscle activity, leading to decreased muscle function.

Joint aging is also closely related to decreased muscle function. Skeletal muscles play a central role in controlling joint stability. Weakened muscle strength around the joint tends to make it susceptible to age-related injuries. The cross-sectional areas of the knee flexor muscle (biceps femoris, suture muscles, thin femoral muscles, semitendinosus, and semimembranosus) all decrease significantly with age. Aging resulted in a remarkable decrease in the tensile strength of the muscles and a considerable decrease in the total cross-sectional area. The decrease in muscle mass due to aging was mainly due to a decrease in the number of muscle fibers and, to lesser extent, to a decrease in muscle fiber area [46]. Decreased muscle function around the joints is a pivotal trigger for periarticular pain, lower extremity weakness, and gait abnormalities. It is also the main contributor to joint instability, cartilage surface stress imbalance, and progressive aggravation of cartilage damage. The decline in total skeletal muscle mass (atrophy) begins at the age of 30–40 years, with lower extremity muscles decreasing at a rate of ~0.7%–0.8%/year. Besides changes in muscle volume, muscle-specific strength (i.e. strength per unit of muscle) decreases dramatically with age, suggesting a decline in muscle strength. At the same time, the body’s physiological functions gradually decline along with age, and as the ROM of the joints decreases, the ability of the muscles to contract is limited, which in turn leads to a further reduction in muscle strength. Additionally, loss of bone mass, decreased bone density, wear and tear of articular cartilage, and joint swelling can also lead to a decrease in muscle strength [47].

Decreased muscle function can also reflect spinal aging. Spinal conditions such as disc degeneration can lead to pain and stiffness in the spine, which negatively impacts muscle strength and endurance, restricting ROM and mobility. This limitation may result in muscle atrophy, which reduces muscle strength [48]. Conversely, decreased muscle function can also lead to structural destabilization of the spine. Spinal stability depends on the structural integrity of the spine and the support of the surrounding muscles. With decreased muscle function, the muscles that maintain spinal stability are unable to effectively support the spine, leading to spinal instability, which accelerates disc degeneration and pathology, and exacerbates pain and motor dysfunction [49].

Accordingly, decreased muscle function may be a potential marker of skeletal aging. Muscle function can be measured directly by plyometric testing, muscle endurance testing, and muscle function assessment, or by imaging methods such as ultrasound (US), dual-energy X-ray absorptiometry (DXA), magnetic resonance imaging (MRI), and other imaging methods to measure muscle thickness, cross-sectional area, and muscle fiber density to assess muscle volume loss.

#### Recommendations

(1) Limited mobility can be a functional marker for predicting skeletal senescence, suggesting skeletal aging (Level A evidence, Class IIa recommendation);
(2) Decreased postural control can be used as a functional marker to predict skeletal aging, suggesting skeletal aging (Level B evidence, Class IIb recommendation);
(3) Decreased muscle function can serve as a functional marker for predicting skeletal senescence, suggesting skeletal aging (Level B evidence, Class IIb recommendation).

### Imaging markers

Owing to its intuitive, accurate, and easy-to-operate characteristics, imaging has been widely used in the diagnosis, efficacy, and prognostic assessment of bone degenerative diseases and age-related diseases. Imaging can dynamically show variations in the structure or composition of skeleton and bone marrow, reveal alterations in joint structure and degeneration of articular cartilage, and provide important clues for the judgment of skeletal aging.

#### Alterations in skeletal structure and composition

##### Decline in bone density

BMD refers to the amount of minerals in bone, and the reduced BMD includes the reduction of bone volume and the loss of bone mineral salts. During aging, parameters such as bone volume fraction, number of trabecular bone, and connection density of cancellous bone decrease dramatically, and the spatial distance of trabecular bone increases; at the same time, increasing age correlates with decreasing phosphate in bone. Commonly used bone density measurement sites include vertebrae and femur, which are important references for whole-body bone density levels. Vertebral BMD decreases with age, with a more rapid decline in women. Bone density has significant clinical value as a marker of skeletal aging and can be used as an early screening indicator of aging in the whole-body skeletal system, which can help early detection and intervention. In addition, BMD can be used as a predictor of fracture risk, which can help to take measures to prevent fractures. Bone density can be assessed by DXA, quantitative computed tomography (QCT), quantitative ultrasound (QUS), and MRI imaging. MRI imaging can reflect the amount of bone mineral salts by the amount of phosphorus in the skeleton [50].

##### Decreased vertebral body height

Bone fractures occur most commonly in the vertebral body due to the decrease in bone mass and bone density associated with aging and are called osteoporotic vertebral compression fractures (OVCFs). OVCFs are particularly common in the elderly. Studies have shown that the prevalence of OVCFs increases with age, with a prevalence of 5% at <60 years of age, 11% at 70–79 years of age, and 18% at ≥80 years of age [51]. In severe cases, spinal deformity results due to compression fractures of multiple segments. Therefore, vertebral height loss without a clear history of violent injury is one of the important biomarkers of spinal aging. Vertebral height loss can be detected by X-ray, CT, MRI, and DXA.

##### Increased bone marrow adipose tissue

Bone marrow adipose tissue (BMAT) is a dynamically changing tissue associated with osteoporosis, bone tumors, etc [52–54]. Different studies have found that studies using 1.5 T magnetic resonance equipment based on chemical shift-encoded water-fat imaging with multiple gradient echoes [55] or T1 Dixon imaging [56] to measure bone marrow fat proportions in parts of the spine (C3–L5), pelvis, ribs, and limb bones have found that age correlates with the spine or trunk bone marrow adiposity ratio correlates with age. Bone marrow fat proportions can be a potential marker for detecting skeletal aging. BMAT percentage, which can be detected by MRI.

##### Bone marrow vascular remodeling

Bone marrow has an abundant vascular system. The red bone marrow consists of blood sinuses and hematopoietic cells. The blood sinusoids are sinusoidal cavities formed after the branching of arterial capillaries entering the red bone marrow. Animal experiments have shown that aging bone marrow experiences an increase of sinusoidal blood vessels and small capillaries, and a decrease of arterioles [57]. Decreased blood flow and vasodilatation in the epiphyses of both ends of the femur in old rats were also found by radiopaque microsphere labeling [58]. Similarly, a significant decrease in proximal femoral blood flow velocity was found in elderly people by Xe-133 isotope labeling [59]. Thus, determination of bone marrow blood flow velocity by isotope labeling imaging may serve as a marker for predicting bone marrow aging.

##### Spinal force line alterations

Spinal force line refers to the distribution and direction of force on the spine, which is determined by a combination of the body’s skeletal structure, muscle tension, ligaments, and other factors. It determines the posture and stability of the body in motion and at rest. The spinal vertebrae may undergo degenerative changes with aging, resulting in deformities in the shape and structure of the vertebrae, including scoliosis, kyphosis, lordosis, and rotation. Spinal deformities are prevalent in individuals over the age of 65, with prevalence rates ranging from 32% to 68% [13]. These alterations may lead to changes in the spinal vertebral lines of force, which are manifested as altered sagittal balance of the spine [60], as well as compensatory postural changes in the pelvis and lower extremities [61]. It was found that with aging, thoracic kyphosis (TK) gradually increased, with a subsequent decrease in lumbar lordosis (LL), and the whole-body sagittal parameters T1 pelvic angle (TPA) and sagittal vertical axis (SVA) gradually increased, whereas the knee flexion angle (KA) and ankle dorsiflexion angle (AA) significantly increase after the age of 50 years, suggesting that additional physiological compensations occur in the lower extremities in maintaining whole-body sagittal equilibrium [61]. Multiple age-related factors have been associated with the development of spinal/joint deformities, including decreased bone density, osteoporosis, spinal degeneration, decreased mobility and balance, and neurodegenerative diseases. Spine/joint deformities can be detected by X-ray, computed tomography (CT), MRI, EOS® whole body standing imaging system, and body photography.

#### Structural changes in the joints

##### Articular cartilage degeneration

Degeneration of articular cartilage is one of the important manifestations of aging, which occurs due to slow cartilage metabolism and poor self-repairing ability [4]. Cartilage degeneration includes degeneration of the intervertebral disc and hyaline cartilage. Degeneration of intervertebral discs mainly manifests as loss of water in the intervertebral discs, loss of height, and atrophy of the nucleus pulposus [62]. The degree of disc degeneration tends to increase with age. In fact, almost 90% of asymptomatic individuals over the age of 60 have a degenerated intervertebral disc [63]. The imaging manifestation of disc degeneration is predominantly T2-like MRI showing decreased water content [64]. In severely degenerated intervertebral discs, reduced intervertebral space height, Schmorl’s nodes, and even vertebral body slippage due to intervertebral space collapse can be detected on X-rays and CT [65]. Further atrophy of the nucleus pulposus and rupture of the annulus fibrosus may occur with gas entering the intervertebral space along the annulus fibrosus fissure, known as the intervertebral vacuum sign, which is seen in advanced degenerative disease.

The imaging manifestations of hyaline cartilage degeneration are primarily loss of cartilage thickness and reduction in cartilage volume. Cartilage thickness in the patella, medial and lateral tibial plateau, and femoral region was negatively correlated with age, and the scores of cartilage defects in the knee joints of all compartments were positively correlated with age, so that the thickness of articular cartilage tissues can be used as one of the bone aging markers. MRI is one of the most used methods for assessing the thickness of cartilage tissue, providing high-resolution images to show the structure of cartilage tissue [66, 67]. In addition, narrowing or asymmetry of the joint space as demonstrated by X-ray, CT, and other examinations can also be used to hypothesize the severity of articular cartilage degeneration.

##### Intra-articular and intervertebral disc mineralization

Intra-articular mineralization (IAM) includes the appearance of calcification within hyaline cartilage, fibrocartilage, meniscus, or the joint capsule [68]. Disc calcification is common in spinal aging, degeneration, and scoliosis. Disc calcification may lead to disc hardening and altered segmental biomechanics, inflammation, and back pain. A population-based study has shown that IAM is associated with knee imaging and age, but not with joint space narrowing or symptom progression in individuals with OA of all ages. However, IAM can predict the progression of OA in people under 60 years old [69]. Therefore, IAM can be used as an evaluative indicator of bone/joint aging. IAM can be detected by X-ray, CT, and MRI scans.

##### The formation of spinal and joint osteophytes

There is a close relationship between the formation of osteophytes in the spine and joints and aging. During aging, the soft tissues around the joints experience continuous pressure, leading to the wearing down and damage of the cartilage. This prompts the growth of extra bone tissue at the edges of the bones, forming lip-shaped bony protrusions known as osteophytes [4]. A study on the other hand found a positive correlation between osteophyte formation and age [70]. Small osteophytes in the knee are very widespread across age and OA risk groups, especially in subjects with no other OA features. This may suggest that these “osteophytes” do not necessarily represent early OA, but rather a physiologic phenomenon associated with aging [71]. Studies of cadaveric spinal osteophytes have also found a 100% incidence of anterior vertebral osteophytes in samples over 40 years of age [72]. These studies suggest that osteophyte formation may be a natural degenerative phenomenon that accompanies the aging process of the human skeleton. Osteophyte formation can be detected by X-ray and CT scans. In recent years, there have also been large population-based studies of hip osteophytes as determined by DXA [73].

##### Cystic degeneration of subchondral bone

Articular cartilage destruction with age, in addition to osteophyte formation, subchondral bone sclerosis, and/or cystic degeneration are prominent pathologic changes. It has been found that subcortical and subchondral bone cystic degeneration, is an important manifestation of skeletal aging and degeneration, and that the incidence of bone cystic degeneration is higher in those presenting with pain [74]. MRI and X-ray are commonly used methods that can detect bone cystic degeneration.

#### Morphologic imaging markers

##### Degenerative spinal curvature

Spinal curvature is a common degenerative spinal deformation phenomenon in middle-aged and elderly people, including axial, coronal, and sagittal deformations of the spine in any combination [13]. Due to skeletal and muscular asymmetry, each vertebra of the spine undergoes a series of degenerative changes. Scoliosis is defined as a lateral curvature of the spine >10°C [75]. Sagittal plane deformities of the spine, most commonly uncompensated kyphosis, typically result in the inability of older adults to maintain normal upright posture and accompanying back pain [76].

Degree of degenerative spinal curvature can be determined from orthostatic and lateral spinal images, and X-ray, CT, or lower radiation DXA can be used to quantify the angle of spinal curvature [76]. Lateral spinal DXA images or lateral X-ray scans can be used to quantify the angle of spinal curvature. Imaging of spinal curvature has the advantage of being precise and easy to follow, and the earlier it is detected and intervened in, the more likely it is that severe pain, compression of nerves, and other serious consequences will be avoided [77].

##### Geometric changes in the hip joint

During aging, the strength of the hip joint is determined not only by bone density but also by the geometry of the hip joint, and it is particularly important to pay attention to its geometric changes. Several longitudinal studies have found that skeletal aging is accompanied by changes in hip joint geometry, such as an increase in cross-sectional area, an increase in bone area, and a decrease in cortical thickness [78, 79]. Cross-sectional analysis of age stratification revealed that aging may be accompanied by a decrease in neck-shaft angle [80, 81]. Multiple studies have found that the shape of the proximal femur is positively associated with hip fracture risk [79]. Therefore, the analysis of the geometry of the hip joint may serve as another important indicator of joint aging and participate in the construction of predictive models for diseases such as hip fracture and hip OA. The geometry of the hip joint can be utilized in medical imaging such as DXA, X-ray, CT, and MRI.

##### Degenerative changes in the knee joint

Knee diameter and cross-sectional area measurements based on knee medical images suggest correlation with extent of cartilage damage [82]. The machine-learning-based algorithm allows for feature extraction of the knee joint and establishing its association with knee pain, knee OA, and total knee replacements [83]. To develop new algorithms for feature extraction using medical images such as DXA, X-ray, CT, and MRI for degenerative changes in the knee joint due to aging, which can help in disease prediction, early intervention, and aging assessment.

#### Molecular imaging markers

##### Markers of bone and cartilage self-composition

Recent advances in imaging technology have made it possible for MRI techniques to noninvasively detect alterations in tissue chemistry. Using MRI, researchers can sensitively distinguish areas of cartilage damage through the glycosaminoglycan (GAG) chemical exchange saturation transfer (gagCEST) technique. The technique was used to measure the concentration of GAG in the cartilage of the knee joints of 21 subjects, and a significant negative correlation was found between CEST values and arthroscopic cartilage (International Cartilage Repair Society) ICRS scores [84], suggesting that this method can be sensitive to detecting changes in GAG concentrations in cartilage tissue. In addition, several studies have found that increase in both the spin-lattice relaxation time constant (T1ρ, sensitive to cartilage proteoglycan content) and transverse relaxation time (T2, sensitive to water, collagen content, and orientation of collagen fibers) under MRI also represent degenerative changes in articular cartilage [67]. All of the above clinical studies reflect articular cartilage degeneration through the detection of cartilage components under MRI, which is not widely practiced in the clinic but can be a potential indicator of joint aging. A small sample study found a significant decrease in the signal intensity of MRI-based correction of ^23^Na in the region of cartilage degeneration [85], which has the potential to be used as a marker of joint aging.

##### Markers of bone and cartilage metabolism

In addition to the detection of the composition of the cartilage itself, different contrast agents based on the metabolic state of the cartilage or bone are reflected as markers of bone or joint degeneration. Studies using positron emission computed tomography/MRI (PET/MRI) in asymptomatic knee populations have found that early metabolic changes in joint aging can be detected not only by high uptake of 18F-NaF, which can visibly detect osteophytes, but also by high uptake of 18F-NaF in the subchondral bone before cartilage degeneration can be observed by MRI [86]. Gd is an anionic contrast agent. During cartilage degeneration, the reduction of negatively charged GAG in the extracellular matrix facilitates the binding of Gd. Therefore, the application of Gd has the potential for the early detection of cartilage degeneration [87], and can be used as a sensitive marker of cartilage aging. In animal experiments, a collagen hybrid peptide has been designed that can specifically bind to and be detected by denatured collagen molecules [88], which provides a new direction for the selection of markers for cartilage or skeletal aging—the establishment of molecules that can be detected by MRI or PET that target damaged Type II collagen or Type I collagen, molecules that can detect extracellular matrix degradation during aging at an early stage. In addition, fluorescent probes based on matrix metalloproteinases (MMPs), Cathepsin K protease, and histone B have been shown to be sensitive to cartilage damage *in vivo* [89], and their application in humans is highly anticipated.

#### Recommendations

(1) Bone mass/bone density can be an imaging marker to predict skeletal aging, and its decline suggests that skeletal aging may be possible (Level A evidence, Class I recommendation);
(2) Decrease in vertebral height (osteoporosis-related fractures) can be an imaging marker to predict bone aging, and its presence suggests possible spinal aging (Level A evidence, Class IIa recommendation);
(3) Bone marrow fat content increases with age and can be used as a marker to predict skeletal aging (Level B evidence, Class IIa recommendation);
(4) Bone marrow blood flow velocity can be used as an imaging marker to predict bone marrow aging, and its slowing suggests possible bone marrow aging (Level B evidence, Class IIa recommendation);
(5) Altered spinal line of force, which can be used as an imaging marker to predict skeletal aging (Level B evidence, Class IIa recommendation);
(6) Cartilage degeneration manifestations in the spine and joints, which can be used as a series of imaging markers to predict skeletal aging (Level A evidence, Class IIa recommendation);
(7) Intra-articular/disc mineralization can be used as an imaging marker to predict skeletal aging, and its increased presence suggests possible skeletal aging (Level B evidence, Class IIb recommendation);
(8) Osteophyte (spinal/articular) formation can be a predictive imaging marker for skeletal aging, and its presence is suggestive of possible skeletal aging (Level A evidence, Class IIa recommendation);
(9) Bone cystic degeneration can be used as an imaging marker to predict skeletal aging, and its increased presence suggests that bone aging may be possible (Level B evidence, Class IIb recommendation);
(10) Muscle cross-sectional area can be used as an imaging marker to predict joint aging, and its decline suggests possible joint aging (Level A evidence, Class IIa recommendation);
(11) Decreased GAG content of articular cartilage or increased T1ρ and T2 signals detected by gagCEST suggests articular cartilage degeneration and joint aging (Level C evidence, Class IIb recommendation);
(12) Increased 18F-NaF uptake in subchondral bone under PET suggests subchondral bone damage and is a potential marker of joint aging (Level C evidence, Class IIb recommendation);
(13) Increased Gd signal under MRI suggests cartilage extracellular matrix degradation, a potential marker of joint aging (Level C evidence, Class IIb recommendation).

### Body fluid markers

Major components of body fluids such as blood, urine, and synovial fluid are indispensable biomarkers for the assessment of skeletal aging due to their noninvasive or minimally invasive nature, high sensitivity, and ease of accurate measurement. The aim of this consensus is to recommend markers related to skeletal aging, and therefore the search strategy for body fluid markers is to screen body fluids for biomarkers that are likely to be highly correlated with the level of skeletal aging. This consensus therefore focuses on aging markers with disease-predictive significance. Skeletal senescence markers change accordingly in various acute injuries or disease interactions and care should be taken to identify them when used in skeletal senescence marker cohort studies.

#### Blood markers

##### Senescence-associated secretory phenotype

Senescent cells secrete factors or release signaling molecules that actively alter the surrounding environment causing senescence to occur in tissues and cells called SASP [90], osteoblasts [3], osteocytes [91], osteoclasts [92], articular chondrocytes [4, 93], intervertebral disc cells [94], and senescent adipocytes in bone marrow all highly express SASP [95]. During skeletal aging, there is a remarkable increase in the expression of inflammatory factors, including c-reactive protein (CRP), interleukin 1 (IL-1), interleukin 6 (IL-6), and tumor necrosis factor-α (TNF-α), etc [96, 97]. Elevated serum inflammatory factors may be a feature of skeletal aging, but they need to be judged in combination with other indicators to differentiate them from elevated inflammatory factors caused by disease or stress conditions.

##### Bone turnover markers

In the process of bone growth and development, the bone turnover rate increases, after reaching the peak bone mass, the bone turnover rate decreases significantly, and thereafter, the bone turnover rate is negatively correlated with the bone density [98]. Intermediate metabolites or enzymes produced during bone turnover are called BTMs. BTMs are categorized into bone formation markers and bone resorption markers. Presently, BTMs that are widely used in clinical diagnosis of osteoporosis include bone formation markers: osteocalcin (OC), N-terminal propeptide of Type I procollagen (P1NP), bone-specific alkaline phosphatase (b-ALP), and other markers; bone resorption markers: C-terminal telopeptide of Type I collagen (CTX-I), amino-terminal cross-linking telopeptide of Type I collagen (NTX-1), deoxypyridinoline (DPD) and tartrate-resistant acid phosphatase 5b (TRAcP5b), and other markers [99–102]. The decrease in markers of bone formation in the blood and the increase in markers of bone resorption in the blood are typical features of skeletal aging and may be considered as body fluid markers for the assessment of skeletal aging. In addition, the greater value of BTMs lies in the dynamic monitoring of an individual (including baseline data, monitoring, or changes over the course of therapy), which facilitates the assessment of the impact on bone turnover rates when medications are used to treat osteoporosis or other metabolic bone diseases. Notably, secondary osteoporosis or other metabolic bone diseases such as hyperparathyroidism and malignant tumor bone metastases can also result in altered BTMs [103, 104]. Consequently, the combined testing of the above-secreted factors associated with osteogenesis and osteoresorption can be considered as a body fluid marker for the assessment of skeletal aging, but its use should be analyzed in conjunction with clinical synthesis.

##### Hormones and related proteins

Vitamin D is a steroid derivative. Vitamin D2 obtained from food and vitamin D3 obtained by irradiation of the skin with ultraviolet light (wavelength 290–320 nm) binds to vitamin D-specific binding proteins after entering the bloodstream, which are then transported to the liver, where they are formed into 25-hydroxyvitamin D (25OHD) by 25-hydroxylase and further converted to biologically active 1,25(OH)2D by 1α-hydroxylase in the kidney to exert their biological functions. Aging and aging-related diseases may lead to decreased synthesis or impaired metabolism of active vitamin D. Vitamin D deficiency and chronic negative calcium balance can lead to secondary hyperparathyroidism, increased secretion of parathyroid hormone (PTH), active osteoclasts, and increased bone resorption, which can cause or exacerbate osteoporosis. Vitamin D, as an important factor in promoting calcium absorption, is also thought to be related to osteoporosis. A survey of postmenopausal women found that low serum levels of vitamin D were associated with a higher incidence of osteoporosis [105], but another survey of older men did not find a similar correlation [106]. Therefore, serum vitamin D levels may only be suitable for use as a predictor of skeletal aging in women. Vitamin D, while regulating calcium and phosphorus metabolism and maintaining normal bone mineral salts in the body, also plays an important role in the maintenance of muscle health, and is closely associated with the development of sarcopenia [107], and as mentioned above, decreased muscle mass and decreased muscle function can also be used as imaging and functional markers for the prediction of skeletal aging. Therefore, serum A and PTH levels can likewise be considered as body fluid markers for the assessment of skeletal aging. Similarly, the above markers should be used for assessment with care to exclude clinical conditions that would cause them to be altered, such as hyperparathyroidism.

The most fundamental changes in human aging are hormonal changes, with total testosterone declining slowly with the aging process in men and estrogen production retreating rapidly after menopause in women [108, 109]. In fact, before menopause, although estrogen levels are normal, follicle-stimulating hormone (FSH) and luteinizing hormone (LH) have risen, indicating that ovarian follicular function has gradually decreased. FSH and LH levels are higher in postmenopausal women than in premenopausal women [110]. The changes in sex hormone secretion and its regulatory mechanisms that accompany reproductive aging not only lead to a decline in reproductive function, but are also associated with various clinical symptoms of aging, including skeletal aging.

Estrogen deficiency as an important pathogenesis of primary osteoporosis [111]. Decreased estrogen levels reduce the inhibitory effect on osteoclasts, leading to increased bone resorption, although osteoblast-mediated bone formation is also increased, but not enough to compensate for excessive bone resorption. On the other hand, aging and estrogen deficiency result in persistent hypoactivation of the immune system and a pro-inflammatory state. Inflammatory mediators, such as TNFα, IL-1, IL-6, IL-7, IL-17, and prostaglandin E2 (PGE2), induce the expression of M-CSF and RNAKL, which stimulate osteoclasts, resulting in bone loss. A large amount of evidence-based medical evidence shows that menopausal hormone therapy can effectively reduce bone loss and the risk of vertebral, non-vertebral, and hip fractures in postmenopausal women, and the therapeutic efficacy is certain [112]. Testosterone affects bone metabolism in males by stimulating osteogenesis and inhibiting osteoclastogenesis directly via the androgen receptor, and indirectly as a precursor of estrogen, which is converted to estrogen by the enzyme aromatase. Therefore, the sex hormones estrogen, testosterone, FSH, and LH can also be considered as humoral markers for the assessment of skeletal aging.

Insulin-like growth factor 1 (IGF-1), which is produced by growth hormone stimulation secreted by the pituitary gland, is an important growth factor that promotes bone growth and development [113]. Systemic and skeletal levels of IGF-1 decline substantially with age in humans [114, 115]. Systemic IGF-1 level was also decreased in aged mice [116]. Conditional deletion of *Igf1* in bone marrow caused bone loss both in adult and aged mice [117]. Clinical studies have shown that systemic and bone marrow localized IGF-1 levels significantly decrease with advanced age. Therefore, systemic or bone marrow localized IGF-1, can be considered as a blood marker for assessing skeletal aging. In summary, 25OHD, PTH, sex hormones, and IGF-1 can be considered as blood markers for assessing skeletal aging.

##### Matrix proteins

Chondrocytes maintain cartilage elasticity by secreting extracellular matrices including proteoglycans and Type II collagen. During aging, superficial cartilage precursor cells senesce and are exhausted, chondrocytes are reduced, and the ability to synthesize extracellular matrix is impaired. Proteins responsible for extracellular matrix metabolism-related proteins enter the bloodstream from the joint cavity and are expected to serve as markers characterizing cartilage status.

Sustained low-dose inflammation due to aging can lead to increased synthesis of cartilage oligomeric matrix protein (COMP). It was shown that patients with OA of the knee have relatively elevated serum levels of COMP. Therefore, serum COMP levels may serve as one of the markers of joint aging [118]. It is important to note that elevated COMP levels may be associated with a variety of diseases that cause cartilage destruction, such as rheumatoid arthritis, psoriatic arthritis, and intervertebral disc degeneration, and therefore raised serum COMP levels should be analyzed in conjunction with the clinical picture as a marker of joint aging.

MMPs are proteases involved in the degradation of the extracellular matrix. MMP-13 is the main MMP involved in cartilage degradation, which cleaves Type II collagen [119]. The level of MMP-13 in serum samples from patients with OA of the knee was negatively correlated with the volume of knee cartilage and significantly positively correlated with the volume of cartilage defects, suggesting that serum MMP-13 could be a clinically useful biomarker for diagnosing OA of the knee [120]. The expression of MMP-13 is elevated in cartilage tissues of aged joints in the absence of structural destruction of cartilage tissue, suggesting that serum MMP-13 is expected to serve as a potential marker of skeletal aging in articular cartilage.

##### Additional potential blood markers

Hematological factors that are closely associated with skeletal aging also include, grancalcin (GCA) secreted by immune cells [121, 122], reticulocalbin 2 (RCN2) [121], and semaphorin 3A (SEMA3A) [123], among others. Among them, GCA inhibits bone formation by promoting adipogenesis, and inhibition of GCA can alleviate skeletal aging [121]. RCN2 promotes bone formation by promoting lipolysis, and supplementation of RCN2 recombinant proteins can alleviate load loss as well as age-related bone loss [121]. Human serum levels of SEMA3A increase with age and decrease significantly after female menopause, and in a mouse animal model, SEMA3A deficiency in osteocytes leads to severe bone reduction in aged mice [123]. All of these molecules are expected to serve as specific markers for assessing the level of skeletal aging in the skeletal system, but there is a lack of more relevant clinical evidence to consider them as candidate markers of bone aging for validation in subsequent cohort studies.

#### Synovial fluid markers

Joint aging is accompanied by synovitis, and the corresponding histologic changes include synovial thickening, macrophage and lymphocyte infiltration, and blood vessel formation. In aging synovial tissues, the number of M1-type macrophages increases and releases pro-inflammatory cytokines, such as IL-6, which is expected to serve as a marker of bone and joint aging [124–126]. However, IL-6 is easily disturbed by trauma, and it is important to exclude trauma when making a diagnosis. Synovial fluid lubricates the joints while acting as a mediator to transport nutrients to the cartilage and carry out metabolites. Analysis of a large amount of osteoarthritic synovial fluid proteomic data in recent years found that presenilin 1 (PSN1) [127], lysine-specific demethylase 2B (KDM2B) [128], tenascin C (TNC) [129], etc. change with body age, but all lack large-scale clinical studies to confirm that they may reflect the process of joint aging and degeneration.

#### Urine markers

Glucosyl-galactosyl pyridinoline (Glc-Gal-PYD) is an irreducible cross-link of the collagen molecule that serves as a specific marker of synovial pathological activity and can be released from synovial tissues but is virtually absent from bone, cartilage, and other soft tissues [130–132]. High levels of Glu-Gal-PYD in urine are associated with increased cartilage loss. Animal experiments revealed significant spontaneous synovial hyperplasia with inflammatory infiltration in aged mice, suggesting that increased urinary Glc-Gal-PYD could potentially be used to characterize joint aging.

CTX-I and NTX-I are degradation products of Type I collagen, and C-terminal crosslinked telopeptide of Type II collagen (CTX-II) is a degradation product of Type II collagen. The concentrations of CTX-I, NTX-I, and CTX-II were increased in the urine of patients with OA of the knee, but in a Japanese cohort study, there was no significant difference in the NTX-I levels of the different OA grade groups, whereas there was a significant difference in CTX-II [133, 134].In conclusion, CTX-II levels in urine can be considered as a marker for assessing skeletal aging and can be further validated in subsequent cohort studies.

#### Gut microbiota

Microbiota changes contribute to skeletal aging and pathological bone loss [135]. Alterations in the consistency of the gut flora in osteoporosis patients are characterized by an increase in the abundance of *Enterococcus faecalis* (*E. faecalis*) spp. and *Streptococcus* spp. and a significant decrease in the abundance of *Bifidobacterium* spp. and *Prevotella* spp. These altered microbial compositions may influence the process of skeletal aging by affecting the absorption of nutrients such as cholecalciferol and vitamin D, GAG degradation, and amino acid metabolism in osteoporosis patients [136]. The relative abundance of *Streptococcus*, *E. faecalis*, *Clostridium*, *Cholera*, and *Desulfovibrio* spp. was higher in OA patients, and *Rochesteria* spp. and *Ruminalococci* spp. were less abundant, compared with gut microorganisms in OA patients with knee OA and in the healthy population. *E. faecalis* spp. and *Clostridium* spp. were positively associated with articular cartilage degeneration in mice, and increased *Bifidobacterium* spp. were found to reduce cartilage damage and Type II collagen degradation in osteoarthritic mice.

All of the above flora ratio assays are expected to serve as specific markers for assessing the level of skeletal system aging, but direct evidence that can characterize skeletal aging is lacking, and they can be considered as candidate markers of skeletal aging for validation in subsequent basic studies as well as clinical cohort studies.

#### Recommendations

(1) Bone turnover markers (BTMs) in serum (P1NP, OC, b-ALP, etc.) and BTMs in urine (β-CTX, NTX, TRAP5b, DPD, etc.) can be considered as predictive markers of skeletal aging, and changes in BTMs are suggestive of possible bone marrow aging (Level A evidence, Class IIa recommendation).
(2) 25OHD, PTH, sex hormones, and IGF-1 in serum can be considered as predictive markers of skeletal aging, with regular expression alterations suggesting that bone marrow aging may be possible (Level B evidence, Class IIb recommendation);
(3) Serum COMP and MMP-13 are considered as markers for predicting skeletal aging, with decreased concentrations suggesting that joint aging may be possible (Level B evidence, Class IIb recommendation);
(4) Serum-secreted proteins such as GCA, RCN2, and SEMA3A can be considered as markers for predicting skeletal aging, and their decreased expression suggests possible skeletal aging (Level C evidence, Class IIb recommendation);
(5) Urine Glc-Gal-PYD, CTX-II can be considered as markers for predicting skeletal aging, and its elevation can be a marker for predicting joint aging (Level B evidence, Class IIb recommendation);
(6) Synovial fluid in synovial membrane with M1-type macrophages and IL-6 can be considered as markers to predict joint aging. Elevated cell counts of M1-type macrophages and increased expression of IL-6 suggest that joint aging may be possible (Level B evidence, Class IIb recommendation);
(7) The abundance of commensal gut microbiota (*E. faecalis* spp. and *Streptococcus* spp.) in feces can be considered as a predictor of skeletal aging, with elevated abundance suggesting possible skeletal aging (Level C evidence, Class IIb recommendation).

## Assessment of skeletal aging, construction of prediction models

Skeletal aging involves a full range of changes in bone from molecular to functional, and thus accurate evaluation of the state of skeletal aging requires the integration of multidimensional and multiscale information. Significant progress has been made in recent years in exploring the biological age of bone (bone clock). Measuring the biological age of the human skeleton is important for developing effective intervention strategies for skeletal aging that address the drivers of skeletal aging.

In order to answer the fundamental scientific question of “how old is bone now,” bone clock is the best approach. Bone clock is a measuring tool of the biological age based on human physiology and anatomy, accurately reflecting the actual state of the human body’s tissue structure and physiological functions. Bone clock can provide a more accurate quantitative index for assessing the aging state of the human body. Skeletal age has a relatively early start compared with the age of other organs, and the early applications of skeletal age are mainly in the fields of detecting the growth and development level of children, determining the age and qualification of athletes, and forensic identification [137, 138]. The morphological characteristics of bones are inconsistent at different periods of skeletal development, so the X-ray method was adopted to measure bone age by detecting the left carpal bones of Chinese people. Assessing the difference between bone age and actual age reflects whether an individual is mature or has delayed/premature aging, but individual factors (e.g. lifestyle, health, and nutrition) can affect bone remodeling, resulting in a less accurate and more difficult estimation of bone age in adulthood [137]. Complementary studies in related fields (e.g. bone molecular, histomorphometry) have the potential to provide new approaches and improve existing age estimation methods. Skeletal aging can lead to the development of a variety of degenerative pathologies, such as osteoporosis, OA, intervertebral disc degeneration, and sarcopenia. Accurate analysis of bone clock in adulthood is expected to aid in the prediction and prevention of diseases associated with skeletal aging. It should be noted that due to the lack of specificity in the prediction of bone clock, it cannot be used as an independent predictor of disease and needs to be combined with disease-specific markers.

Natural population cohorts are the best research model to clarify disease risk factors. Currently, numerous aging-related research cohorts have been established worldwide [139–145].These studies have collectively contributed to global aging research and also simultaneously promoted multi-organ aging research. Presently, there are also several research cohorts conducted around skeletal degenerative diseases in China, such as a large-scale cohort study of OA conducted at the Shanghai Sixth People’s Hospital, which found that glucagon-like peptide-1 (GLP-1) receptor agonists can contribute to effective weight loss, improve symptoms in patients with knee OA, and reduce the rate of knee surgeries [146]; and an osteoporosis-related cohort study conducted by Xiangya Hospital demonstrated that denosumab can effectively improve glucose metabolism [147]. However, there are relatively few cohort studies directly related to skeletal aging, and there is no well established and recognized skeletal aging assessment and early warning model in the world.

The development of aging assessment has been accelerated with the rise of high-throughput sequencing, the construction of cloud platforms, and big data analysis. Genome-wide association studies, whole blood genomes, conventional transcriptomes, non-coding RNAs, DNA methylation, and proteomics are gradually being incorporated into the field of aging [148]. As emerging sequencing tools such as single-cell sequencing technology and spatial transcriptome sequencing mature, it is highly likely that they will be incorporated into aging cohort studies in the future. The large amount of data and complexity of the assessment metrics that are incorporated result in skeletal aging assessments becoming expensive and difficult to generalize. The geographic locations of population-based cohort studies, the collection indicators developed by individual medical institutions, the methods used to collect data, and the algorithms used for data analysis vary to some extent, leading to heterogeneity among databases, reducing study compatibility, increasing barriers to scientific research, and limiting the generalizability and scientific value of the data generated by a single center. It is crucial to establish uniform criteria for the inclusion of skeletal aging in the assessment and to standardize the principles of data management.

## Conclusion and future perspectives

### Recommendation of skeletal aging biomarkers in focus

Based on the discussion among experts, skeletal aging biomarkers are recommended from three dimensions: skeletal function, imaging, and body fluids. These markers include tests that reflect changes in limb mobility, postural control, muscle function, imaging changes in the structure and composition of bones and joints, as well as alterations in the composition of body fluids, such as blood, urine, and synovial fluids. Future focus should be on validating these biomarkers in different age groups, as shown in Table 2. We have appended the abbreviations in this consensus and their full names in Table 3.

**Table 3.** Abbreviations in this consensus

Abbreviations	Full name of abbreviation
25OHD	25-hydroxyvitamin D
AA	Ankle dorsiflexion angle
ABC	Aging biomarker consortium
b-ALP	Bone-specific alkaline phosphatase
BMAT	Bone marrow adipose tissue
BMD	Bone mineral density
BMI	Body mass index
BTMs	Bone turnover markers
COG	Center of gravity
COMP	Cartilage oligomeric matrix protein
COP	Center of pressure
COR	Class of recommendation
CROM	Cervical range of motion
CRP	c-reactive protein
CTX-1	C-terminal telopeptide of type I collagen
CTX-II	C-terminal crosslinked telopeptide of Type II collagen
DPD	Deoxypyridinoline
DXA	Dual-energy X-ray absorptiometry
gagCEST	Glycosaminoglycan chemical exchange saturation transfer
GCA	Grancalcin
Glc-Gal-PYD	Glucosyl-galactosyl pyridinoline
GLP-1	Glucagon-like peptide-1
IAM	Intra-articular mineralization
ICF	International Classification of Functioning, Disability and Health
IGF-1	Insulin-like growth factor 1
IL-1	Interlenkin 1
IL-6	Interlenkin 6
JPS	Joint position sense
KA	Knee flex angle
KDM2B	Lysine-specific demethylase 2B
LL	Lumbar lordosis
LOE	Level of evidence
MMPs	Matrix metalloproteinases
MRI	Magnetic resonance imaging
NTX-1	Amino-terminal cross-linking telopeptide of Type I collagen
OA	Osteoarthritis
OC	Osteocalcin
OVCFs	Osteoporotic vertebral compression fractures
P1NP	N-terminal propeptide of Type I procollagen
PS1	Presenilin 1
PET	Positron emission computed tomography
PTH	Parathyroid hormone
QCT	Quantitative computed tomography
QUS	Quantitative ultrasound
RCN2	Reticulocalbin 2
ROM	Range of motion
SASP	Senescence-associated secretory phenotype
SEMA3A	Semaphorin 3A
SVA	Sagittal vertical axis
TK	Thoracic kyphosis
TNC	Tenascin C
TNF-α	Tumor necrosis factor-α
TPA	T1 pelvic angle
TRAcP5b	Tartrate-resistant acid phosphatase 5b
US	Ultrasound

### The working route map of our skeletal aging biomarker research

Through the expert consensus, these markers will be further validated in population cohorts in the future to promote high-quality population studies of skeletal aging markers. The translational link between basic and clinical research will be established to accelerate the research process of skeletal aging intervention and contribute to healthy aging of human skeleton.

The framework of China’s skeletal aging marker research includes: (i) promote the standardization and expansion of cohort construction of China’s “Large-Sample Large-Region Skeletal Aging Research Program”; (ii) to establish a network of bone aging markers suitable for the Chinese population, to find the inflection points of bone aging, and to define the time points for intervention; (iii) promote the development of biomarker detection technology and establish an artificial intelligence-based skeletal age measurement system and disease prediction model; (iv) combine industry, academia, and research to promote the establishment and application of research guidelines and standards for bone aging markers in China.
